# Usefulness of intraoperative Indocyanine green video angiography to select the recipient artery for bypass surgery in arteriosclerotic occlusion of the middle cerebral artery: a technical case report

**DOI:** 10.1186/s41016-018-0129-4

**Published:** 2018-07-16

**Authors:** Hiroaki Matsumoto, Yasuhisa Yoshida

**Affiliations:** Department of Neurosurgery, Cerebrovascular Research Institute, Eishokai Yoshida Hospital, Daikai-dori9-2-6, Hyogo-ku, Kobe, 652-0803 Japan

**Keywords:** Indocyanine green video angiography, Recipient artery, Superficial temporal artery-middle cerebral artery bypass

## Abstract

**Background:**

In superficial temporal artery-to-middle cerebral artery (STA-MCA) bypass surgery, indocyanine green video angiography (ICG-VA) is usually used to verify bypass patency. Less-commonly reported is the ability to use this technique to evaluate candidate recipient vessels based on either collateral flow or identification of the distal branch of interest.

**Case presentation:**

An 82-year-old man presented with progressive cerebral infarction due to infarction of the M2 inferior trunk of the right middle cerebral artery. He underwent superficial temporal artery-middle cerebral artery bypass to prevent further ischemia 1 week after the initial stroke. In the surgery, M4 cortical arteries fed by the inferior trunk could not be identified as recipient arteries. Intraoperative ICG-VA showed delayed luminescence of some M4 arteries. Because the M4 arteries fed by the inferior trunk showed delayed retrograde flows from the anterior cerebral artery on preoperative digital subtraction angiography, the M4 arteries with delayed luminescence on ICG-VA were considered to be M4 arteries fed by the inferior trunk and selected as the recipient arteries.

**Conclusions:**

ICG-VA shows differences in flow speed as delayed luminescence. This finding may be useful for detecting target vessels.

## Background

Microscope-integrated near-infrared indocyanine green video angiography (ICG-VA) is a simple, reliable, fast, and noninvasive technique introduced into neurosurgery for intraoperative observation and documentation of blood flow [1–5]. In superficial temporal artery-to-middle cerebral artery (STA-MCA) bypass surgery, ICG-VA is usually used to verify bypass patency. Less-commonly reported is the ability to use this technique to evaluate candidate recipient vessels based on either collateral flow or identification of the distal branch of interest. Most such reported cases involve aneurysmal surgery that requires parent artery occlusion under bypass protection [1–5]. There are few such reported cases of bypass surgery for arteriosclerotic occlusion.

A case of progressive cerebral infarction due to arteriosclerotic occlusion of the M2 inferior trunk of the right MCA, which required STA-MCA bypass to prevent further ischemia, is reported. Although it was difficult to identify the M4 cortical recipient arteries fed by the inferior trunk on gross examination, delayed luminescence on ICG-VA helped identify the correct recipient arteries.

## Case presentation

**Fig. 1:**
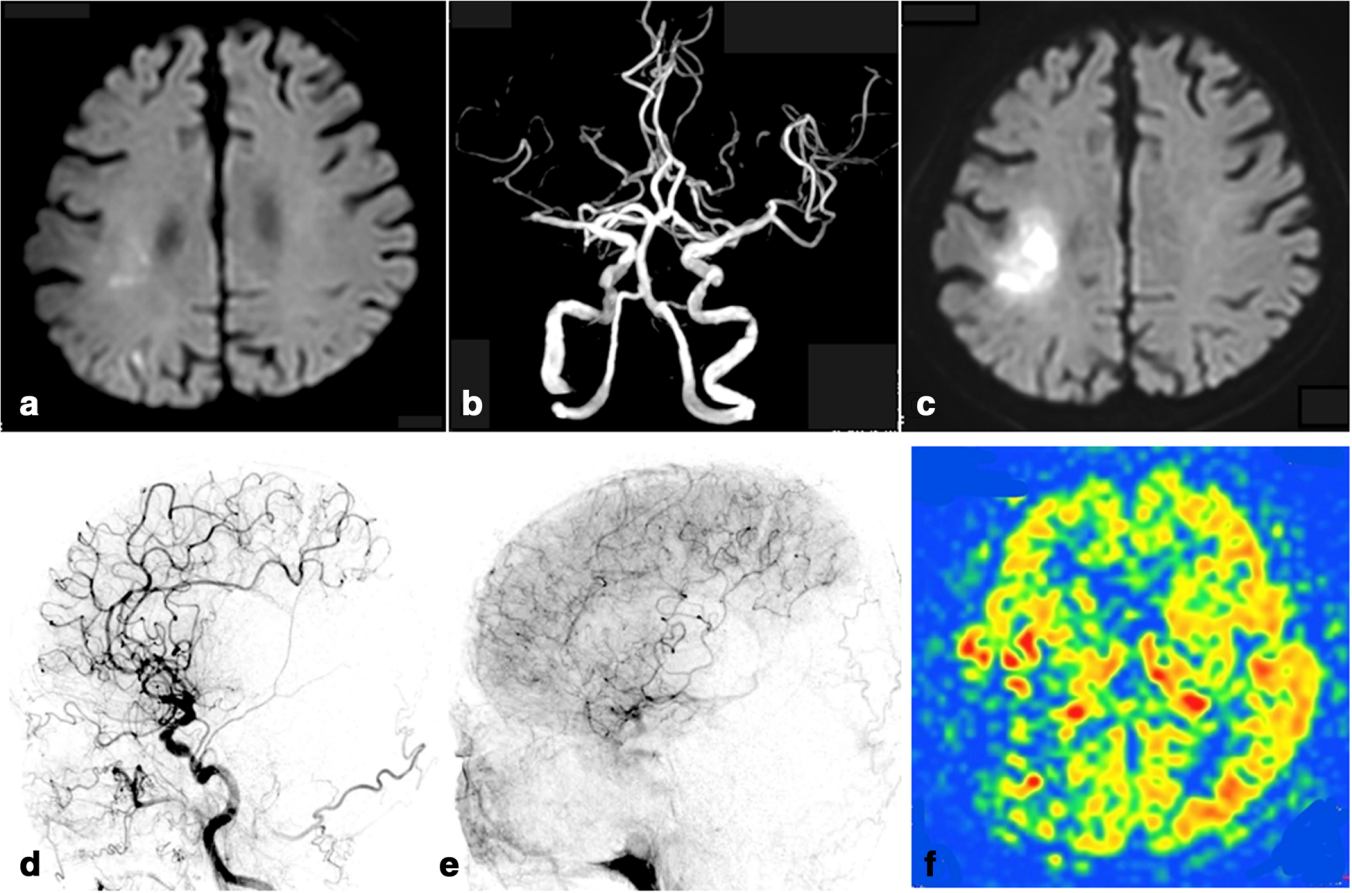
Preoperative investigations. (**a**) Magnetic resonance imaging (MRI) on admission shows a small infarction in the left parietal lobe on diffusion-weighted imaging (DWI). (**b**) MR angiography on admission shows occlusion of the M2 inferior trunk of the middle cerebral artery (MCA). (**c**) Serial MRI shows expansion of cerebral infarction on DWI. D, E: Lateral view of right carotid angiograms. (**d**) In the early phase, there is an avascular area in the territory of the M2 inferior trunk. (**e**) In the late phase, vessels that belong to the M2 inferior trunk are represented by retrograde flow from the anterior cerebral artery. (**f**) Arterial spin labeling (ASL) perfusion imaging showed decreased intensity in the M2 inferior trunk of the right MCA territory

**Fig. 2:**
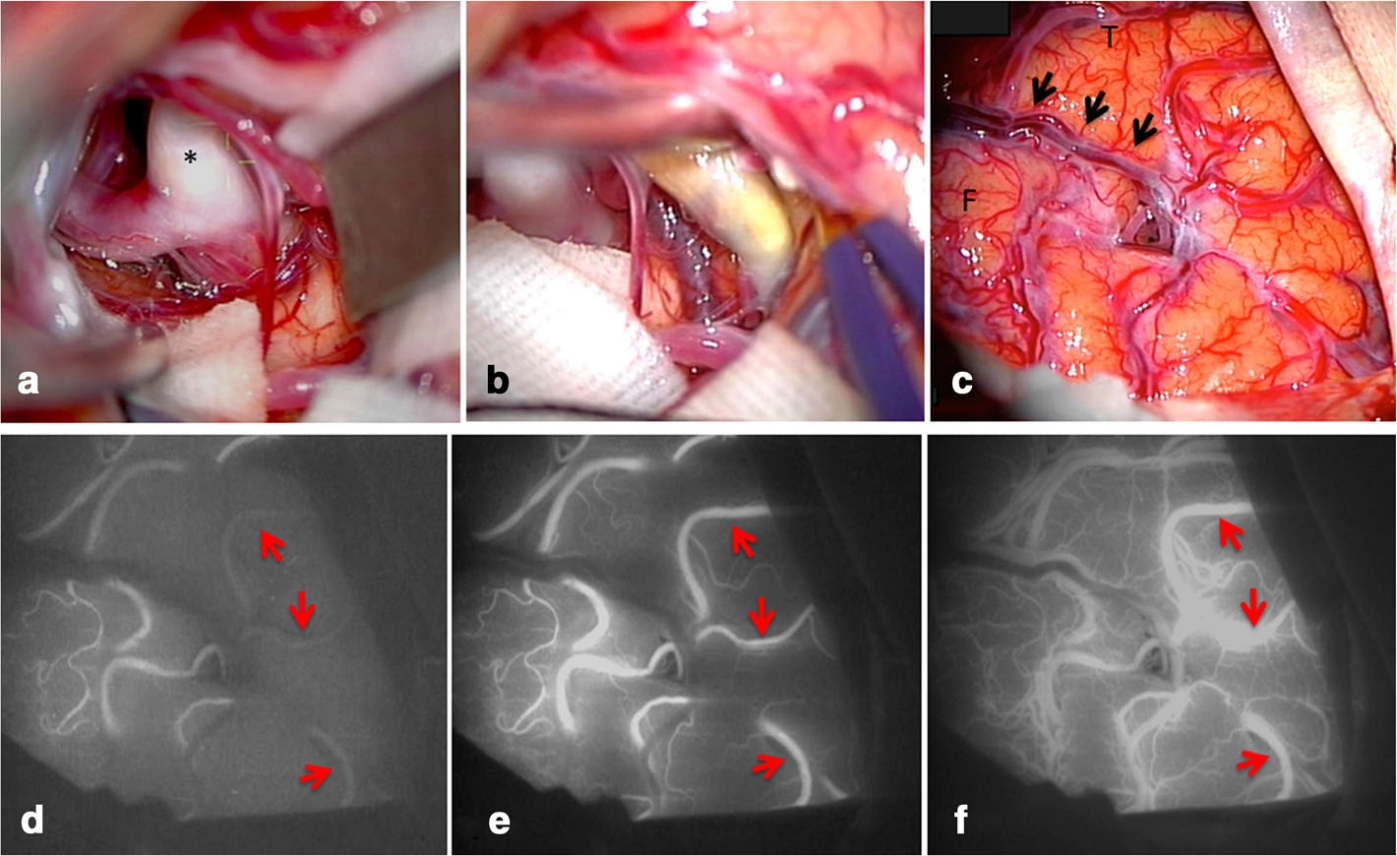
A, B, C: Intraoperative microscopic photographs. (**a**) After dissection of the Sylvian fissure, the M1–2 bifurcation of the middle cerebral artery is exposed (asterisk: M2 inferior trunk). (**b**) After dissection of the M2 inferior trunk from the proximal side, the occluded portion is long. (**c**) There are several M4 cortical arteries on the brain surface (arrows: Sylvian fissure, F: frontal lobe, T: temporal lobe). D, E, F: Intraoperative indocyanine green video angiography in the same view as C. (**d**) In the early phase, M4 cortical arteries fed by the superior trunk show luminescence. (**e**) A short time later, M4 cortical arteries fed by the inferior trunk show luminescence. (**f**) In the late phase, M4 cortical arteries fed by the inferior trunk still show luminescence. (arrows: M4 cortical arteries fed by the inferior trunk)

**Fig. 3:**
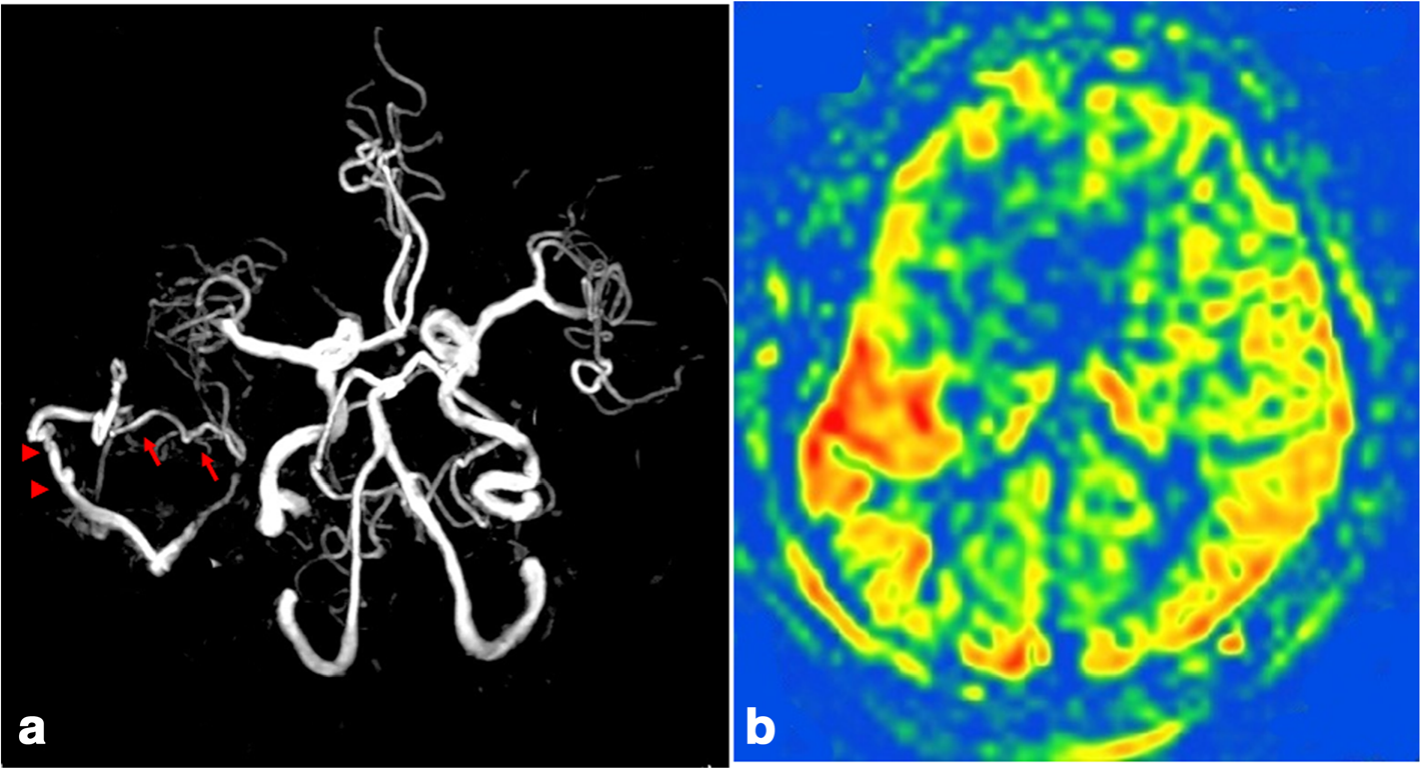
Postoperative investigations. (**a**) Postoperative magnetic resonance angiography showed visualization of the M2 inferior trunk of the right middle cerebral artery (MCA) connected with the superficial temporary artery. (**b**) Postoperative arterial spin labeling perfusion imaging showed increased intensity in the M2 inferior trunk of the right MCA territory

An 82-year-old man presented to our hospital with weakness of the right side of his body. He had a past history of hypertension and gastric cancer. On admission, neurological examination showed mild right hemiparesis (manual muscle testing (MMT) 4). The NIH Stroke Scale (NIHSS) score was 3. Magnetic resonance imaging (MRI) showed a small acute cerebral infarction of the deep white matter in the right parietal lobe (Fig. 1a). MR angiography showed occlusion of the M2 inferior trunk of the right MCA (Fig. 1b). Though he was started on anti-platelet therapy, his left hemiparesis gradually progressed (MMT 2), and his NIHSS score was 7. MRI showed expansion of the cerebral infarction (Fig. 1c). Digital subtraction angiography (DSA) showed occlusion of the M2 inferior trunk of the MCA with retrograde flows from the anterior cerebral artery (ACA) (Fig. 1d, e). Arterial spin labeling (ASL) perfusion imaging showed decreased intensity in the M2 inferior trunk of the right MCA territory (Fig. 1f). Hyperdynamic and induced-hypertension therapies resulted in improvement of his hemiparesis. Progressive cerebral ischemia due to hemodynamic factors was considered, and the patient underwent STA-MCA bypass to prevent progression of cerebral infarction 1 week after the initial stroke. The initial plan involved anastomosis of the M2–3 segment of the inferior trunk. Unexpectedly, the occluded portion was so long that deep dissection of the Sylvian fissure was necessary (Fig. 2a, b). To avoid a deep and narrow site for the anastomosis, the plan was modified to anastomose to the M4 cortical artery instead. However, selecting the recipient arteries was difficult, because it was not obvious which M4 arteries were fed by the inferior trunk in the operative field (Fig. 2c). On intraoperative ICG-VA, the M4 arteries of the inferior trunk showed delayed luminescence (Fig. 2d-f). Thus, two recipient arteries fed by the inferior trunk were selected, and STA-MCA double bypass was performed. After the bypass surgery, the patient showed no further ischemia. Postoperative MR angiography showed visualization of the M2 inferior trunk of the right MCA connected with the STA (Fig. 3a). Moreover, postoperative ASL perfusion imaging showed increased intensity in the M2 inferior trunk of the right MCA territory (Fig. 3b). His hemiparesis gradually improved (MMT4) and he was able to walk with partial assistance.

We obtained consent from the patient for publication of this case report. The case report was approved by the institutional review board of the Eisyokai Yosida Hospital.

## Discussion and conclusions

In selective-targeted STA-MCA bypass, an essential step is to identify the correct recipient artery, because revascularization into a wrong territory is ineffective, with a risk of severe ischemic complications [3]. To target the correct recipient artery, anatomical landmarks, preoperative neuroimaging, neuronavigation, and stereotactic techniques may help the surgeon [3]. However, identification of the correct recipient artery by these methods may be difficult or fail because real-time blood flow cannot be assessed. In the present case, the recipient artery was changed from the M2-M3 segment to the M4 cortical artery intraoperatively. Although the M2-M3 segment could be detected based on anatomical landmarks such as the M1 segment, the M4 cortical arteries fed by the inferior trunk could not be identified on gross examination. To overcome this difficulty, ICG-VA was useful for selecting the recipient artery. The conclusive factor was delayed luminescence. This finding agreed with the preoperative DSA finding of delayed retrograde flows from the ACA, because ICG-VA allows real-time assessment of the cerebral circulation. On the other hand, ICG-VA is used to identify the recipient artery in aneurysmal surgery that needs parent artery occlusion under bypass protection [1–5]. In such cases, under temporary clipping of the proximal vessel, the distal vessel that shows delayed or no filling of luminescence is selected as the recipient. In the present case, ICG-VA was performed under similar conditions, because the M2 inferior trunk was occluded.

On the other hand, it is controversial to perform urgent STA-MCA bypass like our case. However, efficacy of urgent STA-MCA bypass has been reporte [6–8]. We have performed urgent STA-MCA bypass in patients with progressing stroke owing to occlusion of internal carotid artery or MCA based on our own experiences [9]. In this case, we selected urgent STA-MCA bypass because the patient presented progressing stroke in spite of aggressive medication. As a result, the patient showed good recovery.

In conclusion, ICG-VA shows differences in flow speed as delayed luminescence. This finding may be useful for detecting target vessels.

## Abbreviations

ACA: anterior cerebral artery
DSA: digital subtraction angiography
ICG-VA: indocyanine green video angiography
MCA: middle cerebral artery
MMT: manual muscle testing
NIHSS: NIH Stroke Scale
STA: superficial temporal artery
